# 4-(3,3-Dimethyl­perhydro-1,3-oxa­zolo[3,4-*a*]pyridin-1-yl)-2,8-bis­(tri­fluoro­meth­yl)quinoline

**DOI:** 10.1107/S1600536810006562

**Published:** 2010-02-27

**Authors:** James L. Wardell, Solange M. S. V. Wardell, Edward R. T. Tiekink

**Affiliations:** a Centro de Desenvolvimento Tecnológico em Saúde (CDTS), Fundação Oswaldo Cruz (FIOCRUZ), Casa Amarela, Campus de Manguinhos, Av. Brasil 4365, 21040-900 Rio de Janeiro, RJ, Brazil; b CHEMSOL, 1 Harcourt Road, Aberdeen AB15 5NY, Scotland; c Department of Chemistry, University of Malaya, 50603 Kuala Lumpur, Malaysia

## Abstract

An L-shaped conformation is found in the title mol­ecule, C_20_H_20_F_6_N_2_O, the C—C—C—C torsion angle linking the two fused-ring systems being −92.80 (19)°. The oxazole ring adopts an envelope conformation [the N atom lies 0.579 (2) Å out of the plane defined by the remaining atoms], and the piperidine ring has a chair conformation. Supra­molecular chains are found in the crystal structure that are sustained by C—H⋯π and π–π [3.6089 (10) Å] inter­actions.

## Related literature

For information on mefloquine and its derivatives, see: Maguire *et al.* (2006 ▶); Croft & Herxheimer (2002 ▶); Lima *et al.* (2002 ▶); Biot *et al.* (2000 ▶); Roesner *et al.* (1981 ▶); Kunin & Ellis (2007 ▶). For the synthesis of 1,3-oxazolidines, see: Bergmann *et al.* (1953 ▶); Oh *et al.* (2000 ▶); Saba *et al.* (2007 ▶); Page *et al.* (2007 ▶); Kukharev *et al.* (2007 ▶); Delgado *et al.* (1987 ▶). For the biological activity of 1,3-oxazolidines, see: Moloney *et al.* (1998 ▶); Andes *et al.* (2002 ▶); Kumar *et al.* (2009 ▶).
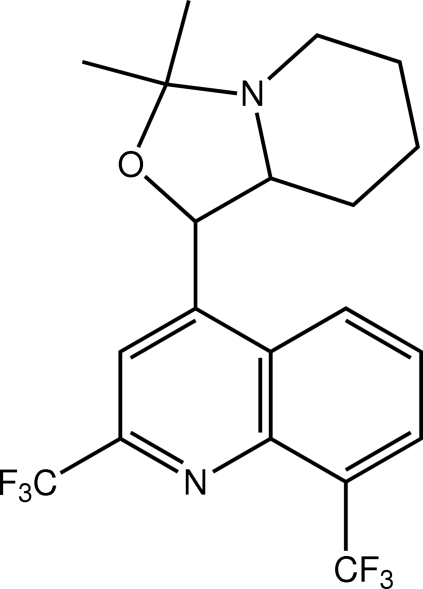


## Experimental

### 

#### Crystal data

C_20_H_20_F_6_N_2_O
*M* *_r_* = 418.38
Triclinic, 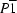

*a* = 8.4192 (3) Å
*b* = 9.1833 (4) Å
*c* = 12.4424 (4) Å
α = 87.912 (2)°
β = 86.666 (2)°
γ = 78.804 (2)°
*V* = 941.78 (6) Å^3^
*Z* = 2
Mo *K*α radiation
μ = 0.13 mm^−1^
*T* = 120 K
0.65 × 0.30 × 0.25 mm

#### Data collection

Nonius KappaCCD area-detector diffractometer
Absorption correction: multi-scan (*SADABS*; Sheldrick, 2007 ▶) *T*_min_ = 0.643, *T*_max_ = 0.746
16907 measured reflections
4275 independent reflections
3107 reflections with *I* > 2σ(*I*)
*R*_int_ = 0.045

#### Refinement

*R*[*F* ^2^ > 2σ(*F* ^2^)] = 0.047
*wR*(*F* ^2^) = 0.141
*S* = 1.06
4275 reflections
264 parameters
H-atom parameters constrained
Δρ_max_ = 0.30 e Å^−3^
Δρ_min_ = −0.36 e Å^−3^

### 

Data collection: *COLLECT* (Hooft, 1998 ▶); cell refinement: *DENZO* (Otwinowski & Minor, 1997 ▶) and *COLLECT*; data reduction: *DENZO* and *COLLECT*; program(s) used to solve structure: *SHELXS97* (Sheldrick, 2008 ▶); program(s) used to refine structure: *SHELXL97* (Sheldrick, 2008 ▶); molecular graphics: *ORTEP-3* (Farrugia, 1997 ▶) and *DIAMOND* (Brandenburg, 2006 ▶); software used to prepare material for publication: *publCIF* (Westrip, 2010 ▶).

## Supplementary Material

Crystal structure: contains datablocks global, I. DOI: 10.1107/S1600536810006562/ez2202sup1.cif

Structure factors: contains datablocks I. DOI: 10.1107/S1600536810006562/ez2202Isup2.hkl

Additional supplementary materials:  crystallographic information; 3D view; checkCIF report

## Figures and Tables

**Table 1:** Hydrogen-bond geometry (Å, °) *Cg* is the centroid of the C4–C9 ring.

*D*—H⋯*A*	*D*—H	H⋯*A*	*D*⋯*A*	*D*—H⋯*A*
C15—H15c⋯*Cg*^i^	0.98	2.87	3.781 (2)	155

Symmetry code: (i) 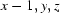.

**Fig. 1.**
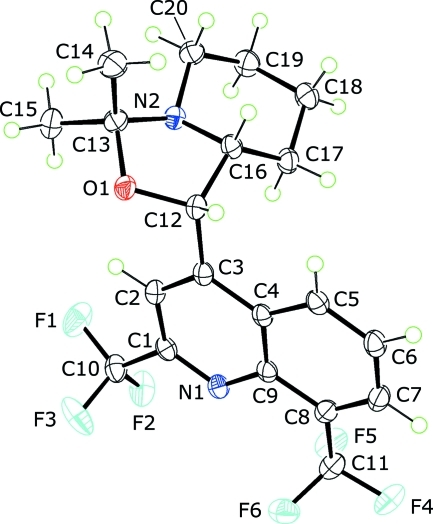
The molecular structure of (I) showing the atom-labelling scheme and displacement ellipsoids at the 50% probability level.

**Fig. 2.**
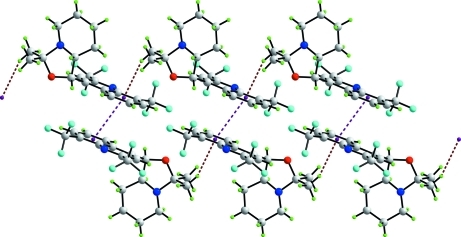
A view of a supramolecular chain in (I) aligned along the a axis. The C–H···π and π–π interactions are shown as brown and purple dashed lines, respectively. Colour code: F, cyan; O, red; N, blue; C, grey; and H, green.

## supplementary crystallographic information

### Comment

Mefloquine, manufactured as the racemic erythro hydrochloride salt is a synthetic analogue of quinine used in the prevention and treatment for malaria in combination with other drugs (Maguire *et al.*, 2006). However, despite its efficacy, this orally-administered drug possesses several important physical and psychological adverse side-effects, such as birth defects, anxiety, aggression, seizures, nightmares, neuropathy, insomnia, central nervous system problems, acute depression, urinary disorders, *etc*. (Croft & Herxheimer, 2002). Due to these effects, mefloquine analogues have been synthesized with the goals of increasing the efficacy and eliminating adverse side-effects (Lima *et al.*, 2002; Biot *et al.*, 2000; Roesner *et al.*, 1981). Mefloquine derivatives are also undergoing tests against other diseases, for example, as anti-viral and anti-tuberculosis agents (Kunin & Ellis, 2007). In the quest for new derivatives, the title compound, 5-[2,8-bis(trifluoromethyl)quinolin-4-yl)-hexahydro-3*H*-oxazolo[3,4-a]pyridine-2-oxaindolizidine (I) has been obtained.

Oxazolidines are frequently prepared from 2-amino-1-hydroxyalkanes and carbonyl compounds (Bergmann *et al.*, 1953; Oh *et al.*, 2000; Saba *et al.*, 2007; Page *et al.*, 2007); in particular cases, azeotropic removal of water or the use of a catalyst is involved (Page *et al.*, 2007). However, problems of stability of oxazolidines and formation of tautomeric mixtures of the oxazolidines and the corresponding imines can limit or complicate this synthetic route (Page *et al.*, 2007). Alternative routes to hexahydro-3*H*-oxazolo[3,4-a]pyridine derivatives include the Hg(OAc)_2_ catalysed cyclization of 2-(vinyloxymethyl)piperidine (Kukharev *et al.*, 2007) and use of 1-naphthalenyl 2-pyridinyl ketone (Delgado *et al.*, 1987). 1,3-Oxazolidine derivatives, in general, have been shown to have useful biological activities (Moloney *et al.*, 1998; Andes *et al.*, 2002; Kumar *et al.*, 2009). The title compound, (I), was isolated unexpectedly from a solution of mefloquine hydrochloride and 2-hydroxybenzoic acid in acetone, followed by initial by recrystallisation from isopropanol, and finally from EtOH.

The molecular structure of (I), Fig. 1, adopts an L-shaped conformation with the quinoline group being approximately orthogonal to the rest of the molecule; for example, the C2–C3–C12–C16 torsion angle is -92.80 (19) °. The five-membered oxazole ring adopts an envelope conformation with the N2 atom lying 0.579 (2) Å out of the plane defined by the remaining atoms. The piperidine ring adopts a chair conformation. The presence of C–H···π, Table 1, and π–π [ring centroid(C4–C9)···ring centroid(C4–C9)^i^ distance = 3.6089 (10) Å for *i*: 1-*x*, 1-*y*, 2-*z*] interactions feature in the crystal packing. Thus, centrosymmetrically related molecules are connected via π–π interactions, and these are linked into a supramolecular chain via C–H···π contacts, Fig. 2.

### Experimental

A mixture of racemic erythro mefloquinium chloride (1 mmol) and 2-hydroxybenzoic acid (1 mmol) in acetone (20 ml) was refluxed for 3 h. The reaction mixture was rotary evaporated and the residue was taken up in isopropanol. Two crops of crystals were collected on maintaining the solution at room temperature. From the second crop, on recrystallisation from EtOH, a small amount of the title compound was obtained, m.p. 424–426 K.

### Refinement

The C-bound H atoms were geometrically placed (C–H = 0.95–1.00 Å) and refined as riding with *U_iso_*(H) = 1.2-1.5*U_eq_*(C).

### Crystal data

**Table:**

C_20_H_20_F_6_N_2_O	*Z* = 2
*M**_r_* = 418.38	*F*(000) = 432
Triclinic, *P*1	*D*_x_ = 1.475 Mg m^−^^3^
Hall symbol: -P 1	Mo *K*α radiation, λ = 0.71073 Å
*a* = 8.4192 (3) Å	Cell parameters from 12184 reflections
*b* = 9.1833 (4) Å	θ = 2.9–27.5°
*c* = 12.4424 (4) Å	µ = 0.13 mm^−^^1^
α = 87.912 (2)°	*T* = 120 K
β = 86.666 (2)°	Block, colourless
γ = 78.804 (2)°	0.65 × 0.30 × 0.25 mm
*V* = 941.78 (6) Å^3^	

### Data collection

**Table:**

Nonius KappaCCD area-detector diffractometer	4275 independent reflections
Radiation source: Enraf Nonius FR591 rotating anode	3107 reflections with *I* > 2σ(*I*)
10 cm confocal mirrors	*R*_int_ = 0.045
Detector resolution: 9.091 pixels mm^-1^	θ_max_ = 27.4°, θ_min_ = 3.0°
φ and ω scans	*h* = −10→10
Absorption correction: multi-scan (*SADABS*; Sheldrick, 2007)	*k* = −11→11
*T*_min_ = 0.643, *T*_max_ = 0.746	*l* = −16→16
16907 measured reflections	

### Refinement

**Table:**

Refinement on *F*^2^	Primary atom site location: structure-invariant direct methods
Least-squares matrix: full	Secondary atom site location: difference Fourier map
*R*[*F*^2^ > 2σ(*F*^2^)] = 0.047	Hydrogen site location: inferred from neighbouring sites
*wR*(*F*^2^) = 0.141	H-atom parameters constrained
*S* = 1.06	*w* = 1/[σ^2^(*F*_o_^2^) + (0.0739*P*)^2^ + 0.249*P*] where *P* = (*F*_o_^2^ + 2*F*_c_^2^)/3
4275 reflections	(Δ/σ)_max_ = 0.001
264 parameters	Δρ_max_ = 0.30 e Å^−^^3^
0 restraints	Δρ_min_ = −0.36 e Å^−^^3^

### Special details

**Table:**

Geometry. All s.u.'s (except the s.u. in the dihedral angle between two l.s. planes) are estimated using the full covariance matrix. The cell s.u.'s are taken into account individually in the estimation of s.u.'s in distances, angles and torsion angles; correlations between s.u.'s in cell parameters are only used when they are defined by crystal symmetry. An approximate (isotropic) treatment of cell s.u.'s is used for estimating s.u.'s involving l.s. planes.
Refinement. Refinement of *F*^2^ against ALL reflections. The weighted *R*-factor wR and goodness of fit *S* are based on *F*^2^, conventional *R*-factors *R* are based on *F*, with *F* set to zero for negative *F*^2^. The threshold expression of *F*^2^ > 2σ(*F*^2^) is used only for calculating *R*-factors(gt) etc. and is not relevant to the choice of reflections for refinement. *R*-factors based on *F*^2^ are statistically about twice as large as those based on *F*, and *R*- factors based on ALL data will be even larger.

### Fractional atomic coordinates and isotropic or equivalent isotropic displacement parameters (Å^2^)

**Table:**

	*x*	*y*	*z*	*U*_iso_*/*U*_eq_	
F1	0.24470 (15)	0.13631 (13)	0.70186 (12)	0.0465 (4)	
F2	0.48941 (14)	0.02882 (12)	0.72734 (10)	0.0376 (3)	
F3	0.31274 (17)	0.05404 (14)	0.85902 (11)	0.0489 (4)	
F4	1.00086 (13)	0.26530 (14)	0.94688 (10)	0.0398 (3)	
F5	0.90320 (13)	0.18513 (14)	0.80980 (9)	0.0369 (3)	
F6	0.80523 (14)	0.14596 (13)	0.96979 (9)	0.0357 (3)	
O1	0.04978 (14)	0.65337 (14)	0.77211 (9)	0.0225 (3)	
N1	0.54732 (17)	0.26383 (16)	0.83292 (11)	0.0209 (3)	
N2	0.13591 (17)	0.69425 (16)	0.59985 (11)	0.0208 (3)	
C1	0.4030 (2)	0.27072 (19)	0.79573 (13)	0.0205 (4)	
C2	0.2882 (2)	0.40134 (19)	0.77803 (13)	0.0208 (4)	
H2	0.1866	0.3973	0.7501	0.025*	
C3	0.3263 (2)	0.53470 (19)	0.80200 (13)	0.0190 (4)	
C4	0.4797 (2)	0.53404 (19)	0.84578 (13)	0.0183 (4)	
C5	0.5296 (2)	0.6653 (2)	0.87684 (13)	0.0221 (4)	
H5	0.4579	0.7585	0.8709	0.027*	
C6	0.6799 (2)	0.6584 (2)	0.91518 (14)	0.0248 (4)	
H6	0.7114	0.7467	0.9360	0.030*	
C7	0.7882 (2)	0.5216 (2)	0.92398 (14)	0.0258 (4)	
H7	0.8934	0.5188	0.9488	0.031*	
C8	0.7437 (2)	0.3925 (2)	0.89724 (13)	0.0222 (4)	
C9	0.5868 (2)	0.3955 (2)	0.85806 (13)	0.0195 (4)	
C10	0.3631 (2)	0.1221 (2)	0.77122 (16)	0.0273 (4)	
C11	0.8617 (2)	0.2472 (2)	0.90593 (15)	0.0280 (4)	
C12	0.2119 (2)	0.67903 (19)	0.77676 (14)	0.0200 (4)	
H12	0.2150	0.7518	0.8341	0.024*	
C13	−0.0137 (2)	0.7020 (2)	0.66828 (14)	0.0225 (4)	
C14	−0.1190 (2)	0.8573 (2)	0.67829 (16)	0.0313 (5)	
H14A	−0.2044	0.8555	0.7351	0.047*	
H14B	−0.1685	0.8881	0.6097	0.047*	
H14C	−0.0516	0.9277	0.6966	0.047*	
C15	−0.1106 (2)	0.5903 (2)	0.63459 (16)	0.0301 (4)	
H15A	−0.0419	0.4911	0.6343	0.045*	
H15B	−0.1486	0.6169	0.5622	0.045*	
H15C	−0.2040	0.5908	0.6854	0.045*	
C16	0.2497 (2)	0.7487 (2)	0.66532 (13)	0.0211 (4)	
H16	0.2174	0.8591	0.6692	0.025*	
C17	0.4194 (2)	0.7103 (2)	0.61295 (14)	0.0248 (4)	
H17A	0.4541	0.6012	0.6094	0.030*	
H17B	0.4965	0.7488	0.6563	0.030*	
C18	0.4196 (2)	0.7800 (2)	0.49901 (15)	0.0290 (4)	
H18A	0.4013	0.8895	0.5037	0.035*	
H18B	0.5269	0.7463	0.4617	0.035*	
C19	0.2881 (2)	0.7372 (2)	0.43406 (15)	0.0289 (4)	
H19A	0.2825	0.7929	0.3643	0.035*	
H19B	0.3165	0.6299	0.4189	0.035*	
C20	0.1239 (2)	0.7705 (2)	0.49433 (14)	0.0251 (4)	
H20A	0.0896	0.8789	0.5035	0.030*	
H20B	0.0420	0.7359	0.4529	0.030*	

### Atomic displacement parameters (Å^2^)

**Table:**

	*U*^11^	*U*^22^	*U*^33^	*U*^12^	*U*^13^	*U*^23^
F1	0.0385 (7)	0.0273 (7)	0.0775 (10)	−0.0082 (5)	−0.0226 (7)	−0.0122 (6)
F2	0.0292 (6)	0.0254 (6)	0.0579 (8)	−0.0043 (5)	0.0073 (5)	−0.0162 (6)
F3	0.0677 (9)	0.0281 (7)	0.0531 (8)	−0.0219 (6)	0.0212 (7)	−0.0005 (6)
F4	0.0252 (6)	0.0483 (8)	0.0446 (7)	0.0006 (5)	−0.0134 (5)	−0.0066 (6)
F5	0.0292 (6)	0.0482 (8)	0.0290 (6)	0.0045 (5)	0.0007 (5)	−0.0115 (5)
F6	0.0369 (7)	0.0323 (7)	0.0348 (6)	−0.0004 (5)	−0.0030 (5)	0.0063 (5)
O1	0.0172 (6)	0.0279 (7)	0.0225 (6)	−0.0056 (5)	0.0007 (5)	0.0008 (5)
N1	0.0202 (7)	0.0227 (8)	0.0199 (7)	−0.0050 (6)	0.0032 (6)	−0.0019 (6)
N2	0.0192 (7)	0.0215 (8)	0.0218 (7)	−0.0043 (6)	−0.0002 (6)	−0.0016 (6)
C1	0.0232 (9)	0.0188 (9)	0.0199 (8)	−0.0063 (7)	0.0041 (7)	−0.0024 (7)
C2	0.0189 (8)	0.0233 (9)	0.0213 (9)	−0.0065 (7)	0.0010 (7)	−0.0026 (7)
C3	0.0192 (8)	0.0228 (9)	0.0158 (8)	−0.0065 (7)	0.0025 (6)	−0.0026 (7)
C4	0.0200 (8)	0.0214 (9)	0.0142 (8)	−0.0068 (7)	0.0026 (6)	−0.0007 (7)
C5	0.0256 (9)	0.0227 (9)	0.0192 (8)	−0.0084 (7)	0.0014 (7)	0.0001 (7)
C6	0.0287 (10)	0.0292 (10)	0.0199 (9)	−0.0138 (8)	−0.0012 (7)	−0.0020 (7)
C7	0.0212 (9)	0.0377 (11)	0.0208 (9)	−0.0109 (8)	−0.0023 (7)	−0.0008 (8)
C8	0.0189 (8)	0.0319 (10)	0.0154 (8)	−0.0048 (7)	0.0011 (6)	0.0002 (7)
C9	0.0211 (8)	0.0247 (9)	0.0138 (8)	−0.0080 (7)	0.0023 (6)	0.0004 (7)
C10	0.0231 (9)	0.0216 (9)	0.0373 (11)	−0.0057 (7)	0.0032 (8)	−0.0038 (8)
C11	0.0220 (9)	0.0368 (11)	0.0244 (10)	−0.0030 (8)	−0.0026 (7)	−0.0046 (8)
C12	0.0186 (8)	0.0210 (9)	0.0213 (9)	−0.0056 (7)	−0.0002 (6)	−0.0032 (7)
C13	0.0208 (9)	0.0229 (9)	0.0230 (9)	−0.0021 (7)	−0.0011 (7)	−0.0013 (7)
C14	0.0270 (10)	0.0282 (11)	0.0354 (11)	0.0014 (8)	0.0035 (8)	0.0004 (8)
C15	0.0240 (10)	0.0369 (11)	0.0315 (10)	−0.0111 (8)	−0.0004 (8)	−0.0041 (9)
C16	0.0230 (9)	0.0202 (9)	0.0214 (9)	−0.0075 (7)	−0.0008 (7)	−0.0018 (7)
C17	0.0216 (9)	0.0297 (10)	0.0242 (9)	−0.0085 (8)	0.0005 (7)	0.0006 (8)
C18	0.0262 (10)	0.0349 (11)	0.0264 (10)	−0.0093 (8)	0.0039 (7)	0.0030 (8)
C19	0.0301 (10)	0.0345 (11)	0.0223 (9)	−0.0077 (8)	0.0025 (7)	−0.0009 (8)
C20	0.0257 (9)	0.0267 (10)	0.0227 (9)	−0.0041 (8)	−0.0025 (7)	−0.0005 (7)

### Geometric parameters (Å, °)

**Table:**

F1—C10	1.341 (2)	C7—H7	0.9500
F2—C10	1.333 (2)	C8—C9	1.429 (2)
F3—C10	1.328 (2)	C8—C11	1.506 (3)
F4—C11	1.346 (2)	C12—C16	1.548 (2)
F5—C11	1.342 (2)	C12—H12	1.0000
F6—C11	1.340 (2)	C13—C15	1.513 (3)
O1—C12	1.434 (2)	C13—C14	1.531 (3)
O1—C13	1.449 (2)	C14—H14A	0.9800
N1—C1	1.315 (2)	C14—H14B	0.9800
N1—C9	1.366 (2)	C14—H14C	0.9800
N2—C16	1.462 (2)	C15—H15A	0.9800
N2—C20	1.465 (2)	C15—H15B	0.9800
N2—C13	1.470 (2)	C15—H15C	0.9800
C1—C2	1.406 (2)	C16—C17	1.518 (2)
C1—C10	1.513 (2)	C16—H16	1.0000
C2—C3	1.372 (2)	C17—C18	1.534 (3)
C2—H2	0.9500	C17—H17A	0.9900
C3—C4	1.429 (2)	C17—H17B	0.9900
C3—C12	1.514 (2)	C18—C19	1.527 (3)
C4—C9	1.419 (2)	C18—H18A	0.9900
C4—C5	1.424 (2)	C18—H18B	0.9900
C5—C6	1.367 (3)	C19—C20	1.516 (3)
C5—H5	0.9500	C19—H19A	0.9900
C6—C7	1.407 (3)	C19—H19B	0.9900
C6—H6	0.9500	C20—H20A	0.9900
C7—C8	1.368 (3)	C20—H20B	0.9900
			
C12—O1—C13	110.26 (13)	C16—C12—H12	109.4
C1—N1—C9	116.60 (15)	O1—C13—N2	101.73 (13)
C16—N2—C20	111.29 (14)	O1—C13—C15	107.83 (15)
C16—N2—C13	105.69 (13)	N2—C13—C15	110.94 (14)
C20—N2—C13	117.78 (14)	O1—C13—C14	109.00 (14)
N1—C1—C2	125.68 (16)	N2—C13—C14	115.09 (15)
N1—C1—C10	114.78 (16)	C15—C13—C14	111.56 (15)
C2—C1—C10	119.54 (15)	C13—C14—H14A	109.5
C3—C2—C1	118.53 (16)	C13—C14—H14B	109.5
C3—C2—H2	120.7	H14A—C14—H14B	109.5
C1—C2—H2	120.7	C13—C14—H14C	109.5
C2—C3—C4	118.39 (16)	H14A—C14—H14C	109.5
C2—C3—C12	120.45 (15)	H14B—C14—H14C	109.5
C4—C3—C12	121.08 (15)	C13—C15—H15A	109.5
C9—C4—C5	118.85 (15)	C13—C15—H15B	109.5
C9—C4—C3	117.96 (15)	H15A—C15—H15B	109.5
C5—C4—C3	123.19 (16)	C13—C15—H15C	109.5
C6—C5—C4	120.61 (17)	H15A—C15—H15C	109.5
C6—C5—H5	119.7	H15B—C15—H15C	109.5
C4—C5—H5	119.7	N2—C16—C17	109.60 (14)
C5—C6—C7	120.52 (17)	N2—C16—C12	100.79 (13)
C5—C6—H6	119.7	C17—C16—C12	119.71 (15)
C7—C6—H6	119.7	N2—C16—H16	108.7
C8—C7—C6	120.74 (16)	C17—C16—H16	108.7
C8—C7—H7	119.6	C12—C16—H16	108.7
C6—C7—H7	119.6	C16—C17—C18	109.14 (15)
C7—C8—C9	120.17 (17)	C16—C17—H17A	109.9
C7—C8—C11	120.07 (16)	C18—C17—H17A	109.9
C9—C8—C11	119.74 (16)	C16—C17—H17B	109.9
N1—C9—C4	122.79 (15)	C18—C17—H17B	109.9
N1—C9—C8	118.15 (16)	H17A—C17—H17B	108.3
C4—C9—C8	119.06 (16)	C19—C18—C17	111.21 (15)
F3—C10—F2	106.77 (16)	C19—C18—H18A	109.4
F3—C10—F1	106.58 (16)	C17—C18—H18A	109.4
F2—C10—F1	106.43 (15)	C19—C18—H18B	109.4
F3—C10—C1	112.06 (16)	C17—C18—H18B	109.4
F2—C10—C1	112.82 (15)	H18A—C18—H18B	108.0
F1—C10—C1	111.76 (16)	C20—C19—C18	111.32 (15)
F6—C11—F5	106.96 (16)	C20—C19—H19A	109.4
F6—C11—F4	106.30 (15)	C18—C19—H19A	109.4
F5—C11—F4	106.15 (14)	C20—C19—H19B	109.4
F6—C11—C8	113.30 (14)	C18—C19—H19B	109.4
F5—C11—C8	112.29 (15)	H19A—C19—H19B	108.0
F4—C11—C8	111.37 (16)	N2—C20—C19	108.88 (15)
O1—C12—C3	109.96 (14)	N2—C20—H20A	109.9
O1—C12—C16	104.86 (13)	C19—C20—H20A	109.9
C3—C12—C16	113.55 (14)	N2—C20—H20B	109.9
O1—C12—H12	109.4	C19—C20—H20B	109.9
C3—C12—H12	109.4	H20A—C20—H20B	108.3
			
C9—N1—C1—C2	1.2 (3)	C7—C8—C11—F5	−115.48 (18)
C9—N1—C1—C10	−178.76 (14)	C9—C8—C11—F5	62.7 (2)
N1—C1—C2—C3	−0.7 (3)	C7—C8—C11—F4	3.4 (2)
C10—C1—C2—C3	179.25 (16)	C9—C8—C11—F4	−178.36 (15)
C1—C2—C3—C4	−1.2 (2)	C13—O1—C12—C3	−122.74 (14)
C1—C2—C3—C12	175.52 (14)	C13—O1—C12—C16	−0.32 (17)
C2—C3—C4—C9	2.4 (2)	C2—C3—C12—O1	24.3 (2)
C12—C3—C4—C9	−174.25 (14)	C4—C3—C12—O1	−159.06 (14)
C2—C3—C4—C5	−177.91 (15)	C2—C3—C12—C16	−92.80 (19)
C12—C3—C4—C5	5.4 (2)	C4—C3—C12—C16	83.81 (18)
C9—C4—C5—C6	1.7 (2)	C12—O1—C13—N2	23.98 (16)
C3—C4—C5—C6	−177.97 (16)	C12—O1—C13—C15	140.73 (14)
C4—C5—C6—C7	0.4 (3)	C12—O1—C13—C14	−98.00 (16)
C5—C6—C7—C8	−1.8 (3)	C16—N2—C13—O1	−39.80 (16)
C6—C7—C8—C9	1.1 (3)	C20—N2—C13—O1	−164.85 (14)
C6—C7—C8—C11	179.30 (16)	C16—N2—C13—C15	−154.28 (15)
C1—N1—C9—C4	0.2 (2)	C20—N2—C13—C15	80.67 (19)
C1—N1—C9—C8	−179.11 (15)	C16—N2—C13—C14	77.87 (18)
C5—C4—C9—N1	178.31 (15)	C20—N2—C13—C14	−47.2 (2)
C3—C4—C9—N1	−2.0 (2)	C20—N2—C16—C17	−64.79 (18)
C5—C4—C9—C8	−2.4 (2)	C13—N2—C16—C17	166.23 (14)
C3—C4—C9—C8	177.30 (14)	C20—N2—C16—C12	168.10 (13)
C7—C8—C9—N1	−179.65 (15)	C13—N2—C16—C12	39.12 (16)
C11—C8—C9—N1	2.1 (2)	O1—C12—C16—N2	−23.53 (16)
C7—C8—C9—C4	1.0 (2)	C3—C12—C16—N2	96.54 (15)
C11—C8—C9—C4	−177.20 (14)	O1—C12—C16—C17	−143.66 (15)
N1—C1—C10—F3	81.59 (19)	C3—C12—C16—C17	−23.6 (2)
C2—C1—C10—F3	−98.4 (2)	N2—C16—C17—C18	58.38 (19)
N1—C1—C10—F2	−39.0 (2)	C12—C16—C17—C18	173.97 (15)
C2—C1—C10—F2	141.05 (17)	C16—C17—C18—C19	−52.9 (2)
N1—C1—C10—F1	−158.85 (15)	C17—C18—C19—C20	52.4 (2)
C2—C1—C10—F1	21.2 (2)	C16—N2—C20—C19	62.59 (18)
C7—C8—C11—F6	123.21 (18)	C13—N2—C20—C19	−175.18 (15)
C9—C8—C11—F6	−58.6 (2)	C18—C19—C20—N2	−55.9 (2)

### Hydrogen-bond geometry (Å, °)

**Table:**

Cg is the centroid of the C4–C9 ring.

**Table:**

*D*—H···*A*	*D*—H	H···*A*	*D*···*A*	*D*—H···*A*
C15—H15c···Cg^i^	0.98	2.87	3.781 (2)	155

Symmetry codes: (i) *x*−1, *y*, *z*.
